# Oral health knowledge, practice and associated factors among Junior High School students of Koforidua, Ghana: a cross-sectional study

**DOI:** 10.1186/s12903-024-04148-2

**Published:** 2024-04-12

**Authors:** Precious Opoku, Samuel Salu, Cyril Kwami Azornu, Joyce Komesuor

**Affiliations:** 1 Public Health Unit, New Juaben North Municipal Health Directorate, Koforidua, Ghana; 2 Department of Epidemiology and Biostatistics, University of Health and Allied Sciences, Hohoe, Ghana; 3 Public Health Unit, Salaga Municipal Hospital, Salaga, Ghana; 4 Department of Population and Behavioral Sciences, University of Health and Allied Sciences, Hohoe, Ghana

**Keywords:** Suggested MeSH terms- schools, Ghana, Oral health

## Abstract

**Background:**

Oral disease poses a significant public health burden for many countries and affects individuals throughout their lifetime, causing pain, disfigurement, impairment of function and reduced quality of life. Among children and adolescents globally, there is a recognized trend of poor oral hygiene, attributed to the accumulation of plaque and calculus deposits that increase with age. This study assessed the knowledge, practice and factors associated with the practice of oral hygiene among Junior High School students (JHS) in Koforidua, in the Eastern Region of Ghana.

**Methods:**

A school-based cross-sectional study design was conducted among 233 JHS students in Koforidua township using a multistage sampling technique. Descriptive and inferential statistics, including frequency, percentage, and Pearson’s Chi-square test, were conducted. The results were interpreted using tables and graphs.

**Results:**

Findings from the study revealed that more than half 126 (54.1%) of the respondents had a good level of knowledge of oral hygiene. The majority 130 (55.8%) of them also had good oral hygiene practice. The findings further indicated that a significant relationship was found between the class or education level of students and good oral hygiene practice (χ2 = 17.36, *p* < 0.001).

**Conclusion:**

Overall, the current study found that over half of the JHS students had good knowledge as well as practice of oral hygiene. This reinforces the significance of oral health education and awareness campaigns, especially in school settings, to improve knowledge, attitudes, and behaviours related to oral hygiene. The study however found only class or education level of students to be statistically associated with the practice of oral hygiene. While other variables did not reach statistical significance, our research serves as a starting point for further investigation and exploration of the various factors that may contribute to oral hygiene practices.

## Background

Maintaining optimal oral health is acknowledged as a fundamental human right [1]. The World Health Organization (WHO) [2] defines Oral Health as “*the state of the mouth, teeth and orofacial structures that enable individuals to perform essential functions such as eating, breathing and speaking and encompasses psychosocial dimensions such as self-confidence, well-being and the ability to socialize and work without pain, discomfort and embarrassment. Oral health varies over the life course from early life to old age, is integral to general health and supports individuals in participating in society and achieving their potential*”. A disease-free oral cavity and its surrounding components are indicators of good oral health [3].

Oral disease poses a significant public health burden for many countries and affects individuals throughout their lifetime, causing pain, disfigurement, impairment of function and a reduced quality of life [4]. Among children and adolescents, there is a recognized trend of poor oral hygiene, attributed to the accumulation of plaque and calculus deposits that increase with age [5]. According to the WHO Global Oral Health Status Report, it is estimated that oral diseases affect close to 3.5 billion people worldwide, with 3 out of 4 people affected living in middle-income countries [6]. An estimated 2 billion people suffer from caries of permanent teeth including 514 million children who suffer from caries of primary teeth globally [6]. Among populations of low socioeconomic status in developing countries, the practice of maintaining oral hygiene may be completely ignored [7]. Consequentially, they have a larger burden of oral diseases, and this link persists throughout the life course, from infancy to old age, independent of a country’s overall economic level [8]. While largely preventable, oral diseases are the most common diseases globally and regionally, affecting an estimated 480 million people (43.7%) in the WHO African Region in 2019 [1].

In Ghana, the situation cannot be underestimated. According to the WHO’s 2019 report, the prevalence of untreated caries in deciduous teeth among children aged 1–9 years was 38.9% [9]. This figure represents a slight decrease from a previous study in 2016, which found the prevalence to be 40.4% [10]. However, the prevalence of severe periodontal disease in people 15 + years (29.3%) remains a major issue, if compared to the previous study which reported prevalence to be 26.7% [11]. Oral hygiene is influenced by both knowledge and behaviour, however, having knowledge about oral health is not enough to ensure good oral hygiene practice [12]. Proper attitude and practice must be adopted [13, 14]. Oral health promotion through schools is recommended by the WHO for improving knowledge, attitude, and behaviour related to oral health and for the prevention and control of dental diseases among school children [15]. Oral health education and a positive attitude towards oral health are both important factors in improving oral care habits [16]. To create an effective oral health education program, it is essential to assess the level of knowledge, attitude, and practice of the population regarding oral health [16, 17]. Hence, the study aimed to assess the knowledge, practice and factors associated with the practice of oral hygiene among JHS students in Ghana.

## Methods

### Study setting, and design

A school-based cross-sectional survey was conducted among students in Junior High Schools in Koforidua. The Koforidua township is located in the New Juabeng Municipality, in the Eastern Region of Ghana. The 2010 Population and Housing Census, put the total population of the Koforidua township at 122,300 which forms 66.6% of the total population in the Municipality. This is made up of 590,567 (48%) males and 632,344 (52%) females. More than 90 per cent (93.3%) of the population in the town live in urban localities. The majority (54.9%) of the study population are in the basic school level, i.e., Primary and Junior High Schools (JHS). A total of 71 JHSs are located in the Koforidua township comprising both public and private schools.

### Study population and participants

All students from JHS 1 to JHS 3 in the Koforidua township were the source of the study population. However, the participants of the study comprised 233 students sampled from five JHSs in the Koforidua township using purposive sampling.

### Inclusion and exclusion criteria

Participation in this study was based on the following criteria;

#### Inclusion criteria

1. Medically fit students.
2. Students who were present in school during the data collection period.
3. Students who had assented to be included in the study.

#### Exclusion criteria

1. Severely ill during the data collection period.
2. Absent from school at the time of data collection.
3. Non-assenting students who did not provide consent to be included in the study.

### Sample size and sampling procedure

Using Cochrane’s single proportion formula, a sample size of 221 was estimated as follows;

$n=\frac{{z}^{2} p(1-p)}{{d}^{2}}$, considering a 5% margin of error, 95% confidence interval = 1.96 and a prevalence of dental caries of 17.4% from a study conducted by [18]. Where;

n = Estimated sample size

*p* = 0.174

q= (1-p).

d = margin of error (0.05)

Z = Test Statistic (1.96)

Accounting for a 5% non-response rate, the total sample size increased to 233.

A multistage sampling procedure was employed in the study. The first stage involved purposive sampling to select a fair representative sample from five JHSs in the Koforidua township. The formula below was used to determine the number of students to be sampled from each of the five selected JHSs based on the total sample size of the study;

$$\eqalign{& {\rm{Number}}\, {\rm{of}}\, {\rm{students}}\, {\rm{from}}\, {\rm{each}}\, {\rm{school}}\, {\rm{to}}\, {\rm{be}}\, {\rm{sampled}} = \cr & {{{\rm{Total}}\, {\rm{sample}}\, {\rm{size}}} \over {{\rm{Number}}\, {\rm{of}}\, {\rm{schools}}}} \cr}$$

$$\eqalign{ & {\rm{Number}}\, {\rm{of}}\, {\rm{students}}\, {\rm{from}}\, {\rm{each}}\, {\rm{school}}\, {\rm{to}}\, {\rm{be}}\, {\rm{sampled}}; \cr & {{233} \over 5}\, = \, 46.6\, \approx \, 47 \cr}$$

Forty-seven (47) students from each of the five JHSs were selected to arrive at a total of 235 students. At the second stage, each class was treated as a separate stratum namely, JHS 1, JHS 2 and JHS 3. Students who met the inclusion criteria in each of the strata were randomly selected using their class register as a sample frame. In the third stage, after the random selection in the second stage, the total number of students exceeded the desired sample size of 233. To arrive at the desired sample size, two students were randomly excluded from the selected sample.

### Data collection

A paper-based questionnaire was used in the present study using a validated questionnaire, adopted from previous studies [12, 19, 20]. The questionnaire was prefaced with an introductory paragraph to clarify the objective of the study, assure anonymous and voluntary participation, and confirm that the responses will be confidential, and accessible only by the authors. It consisted of 27 items divided into 3 parts thus; sociodemographic characteristics, knowledge of oral hygiene and practice of oral hygiene. The questionnaire was pretested on twenty [20] schoolboys in a public school in the Suhum Municipality, of the Eastern Region of Ghana which were not part of the final study. The results obtained from the pre-test were used to make the necessary changes and modifications required in the questionnaire before the final study was carried out. The instrument’s reliability was ensured by performing a Cronbach’s alpha (a) test for internal consistency. The study utilized 20 items in the tool with a scale reliability coefficient (a) value of 0.7.

### Study variables

#### Dependent variable

The dependent variable in the study was the practice of oral hygiene. This was assessed with 6 items on the questionnaire. The items included (I) frequency of brushing (II) how often dental floss is used (III) how often toothbrush is changed (IV) whether fluoridated toothpaste is used for brushing (V) when brushing is done, and (VI) items used in cleaning the teeth. Respondents were allowed to choose from a list of multiple responses. A composite variable was created for practice by assigning a score of “1” to all the positive responses and a score of “0” to all the negative responses for all 6 items. A mean score was generated by adding all these responses. Students who scored below the mean were considered to have “poor practice” and students who scored above the mean were considered to have “good practice” of oral hygiene.

#### Independent variables

The study explored 8 independent variables which were considered in the inferential analysis. The variables included; age, sex, ethnicity, religion, class or education level, occupation of Parents/guardians, level of education of Parents/guardians, and level of knowledge of oral hygiene. All these variables were based on the findings of previous studies adapted from validated questionnaires [12, 19, 20].

### Statistical analysis

The collected data were coded and entered into Epi-Data version 3.1. Data extraction and cleaning were carried out in Excel Sheet and then later exported into STATA V.16.0 (StataCorp. 2019. Stata Statistical Software: Release 16. College Station, TX: StataCorp LLC.) for analysis. To ensure the quality of the data extracted, double entry was done in Epi-Data to address discrepancies which may have occurred during extraction. Descriptive analysis such as simple frequencies and percentages were used to summarize the data. A chi-square test analysis was conducted to assess the association between independent variables and oral hygiene practice. Significant variables were identified at a *p*-value < 0.05.

Logistic regression was not performed because there was no significant association between almost all the variables.

## Results

In all, 233 respondents were involved in the present study with a 100% response rate as presented in (Table 1) below. The mean age recorded was 14.8 ± 1.41 years. The highest occurring age group was 14–16 years. Prominent among these respondents 169 (72.5%) were in JHS 3 and 116 (49.8%) were Akans. Regarding the level of education of their parents/guardians, the majority 97 (41.6%) of them had only a primary level of education and 214 (91.9%) reported that they are Christians.

**Table 1:** Demographic Characteristics of Participants

Variable	Frequency (*n* = 233)	Percentage (%)
**Age group (Years)**		
SD (14.8, ± 1.41)		
11–13 years	38	16.3
14–16 years	169	72.5
17–19 years	26	11.2
**Sex**		
Male	97	41.6
Female	136	58.4
**Ethnicity**		
Akan	116	49.8
Ga/Adamgbe	42	18.0
Ewe	46	19.7
Others	29	12.5
**Religion**		
Christianity	214	91.9
Islam	16	6.9
Traditionalist	3	1.3
**Class or education level**		
JHS 1	63	27.0
JHS 2	69	29.6
JHS 3	101	43.4
**Occupation of Parents/Guardian**		
Farmer	17	7.3
Trader	125	53.7
Government Worker	56	24.0
Others	26	11.2
Unemployed	9	3.9
**Level of education of Parents/Guardian**		
No Formal Education	26	11.2
Primary	97	41.6
Secondary	84	36.1
Tertiary	26	11.2

### Knowledge of students on oral hygiene

Regarding respondents’ knowledge of oral hygiene, the majority of them 125 (53.7%) mentioned ‘not brushing properly’ as a cause of tooth decay. A comparative majority 177 (76.0%) also agreed to the fact that bleeding of gums during brushing is abnormal. Moreover, only 66 (28.3%) said advanced periodontal disease can contribute to heart disease. Predominant among the respondents 99 (42.5%) indicated that chewing tobacco was less harmful to the tooth than smoking a cigarette. Almost all of them 213 (91.4%) confirmed they ever had an education on oral hygiene. (Table 2).

**Table 2:** Knowledge of students on oral hygiene

Variable	Frequency (*n* = 233)	Percentage (%)
**What is the main cause of tooth decay?**		
Bacteria plaque present on the teeth	67	28.8
Not brushing properly	125	53.7
Eating sugary foods	29	12.5
Not going to the dentist	4	1.7
Drinking sodas with sugar	3	1.3
Weak enamel	3	1.3
Drinking sodas without sugar	2	0.9
**Which of the following would prevent your gums from bleeding?**		
Brushing and flossing teeth	64	27.5
Going to the dentist for regular checkups	65	27.9
Eating less sugar	82	35.2
Using toothpaste containing fluoride	22	9.4
**Is bleeding of the gums while brushing your teeth normal?**		
Yes	45	19.3
No	177	76.0
Unsure	11	4.7
**Can advanced periodontal disease contribute to heart disease?**		
Yes	66	28.3
No	105	45.1
Unsure	62	26.6
**Is it normal to lose teeth as one gets older?**		
Yes	139	59.7
No	83	35.6
Unsure	11	4.7
**How often should you visit the dentist?**		
At least once every six months	129	55.4
At least once a year	41	17.6
At least once every two years	4	1.7
When there is a real need	35	15.0
Never	24	10.3
**If one takes proper care of his or her teeth, can they last for a lifetime?**		
Yes	189	81.1
No	32	13.7
Unsure	12	5.2
**My nutrition or daily eating habits affect my oral health**		
Yes	128	54.9
No	94	40.3
Unsure	11	4.7
**Is chewing tobacco less harmful to the tooth than smoking a cigarette?**		
Yes	99	42.5
No	114	48.9
Unsure	20	8.6
**Have you had any education on oral hygiene before?**		
Yes	213	91.4
No	15	6.4
Unsure	5	2.2
**If yes, where?**		
School	172	80.8
Radio/Television	28	13.2
Family	9	4.2
Friends	3	1.4
Others	1	0.5
**How frequently is oral hygiene education given in the school?**		
Daily	31	18.0
Weekly	33	19.2
Monthly	32	18.6
Yearly	76	44.2

### Practice of oral hygiene among students

Table 3 below shows the distribution of respondents’ practice of oral hygiene. The majority 152 (65.2%) of them reported that they brushed their teeth two or more times a day. A comparative majority of them 188 (80.7%) indicated that they use fluoridated toothpaste for brushing. Additionally, most of the respondents 93 (39.9%) reported that they changed their toothbrushes several times a year. More than half of them 127 (54.5%) said they brushed their teeth in the morning before eating and in the night before sleeping. Moreover, a significant majority 192 (82.4%) reported that brushing was initiated by their mothers and 179 (76.8%) it occurred within the ages of 1–5 years.

**Table 3:** Practice of oral hygiene among students

Variable	Frequency (*n* = 233)	Percentage (%)
**How often do you brush your teeth?**		
2 or more times a day	152	65.2
Once a day	76	32.6
Once every 2–3 days	3	1.3
Once every 4–6 days	2	0.9
Once a week	0	0.0
Spontaneously	0	0.0
**How often do you use dental floss?**		
2 or more times a day	77	33.1
Once a day	75	32.2
Once every 2–3 days	11	4.7
Once every 4–6 days	6	2.6
Once a week	22	9.4
Spontaneously	42	18.0
**How often do you change your toothbrush?**		
Once a year	38	16.3
2–3 times a year	90	38.6
Several times a year	93	39.9
Never	12	5.2
**Do you use fluoridated toothpaste for brushing?**		
Yes	188	80.7
No	34	14.6
Unsure	11	4.7
**When do you do your brushing?**		
After eating	14	6.0
Morning before eating	65	27.9
Morning after eating	5	2.2
Morning before eating and night before bed	127	54.5
Morning after eating and evening before bed	22	9.4
**Who initiated brushing?**		
Mother	192	82.4
Father	29	12.5
Others	12	5.2
**At what age was brushing initiated?**		
1-5 Years	179	76.8
> 5 years	15	6.4
Don’t know	39	16.7
**What do you use in cleaning your teeth?**		
Toothpaste and brush	203	87.1
Others	30	12.9

**Fig. 1:**
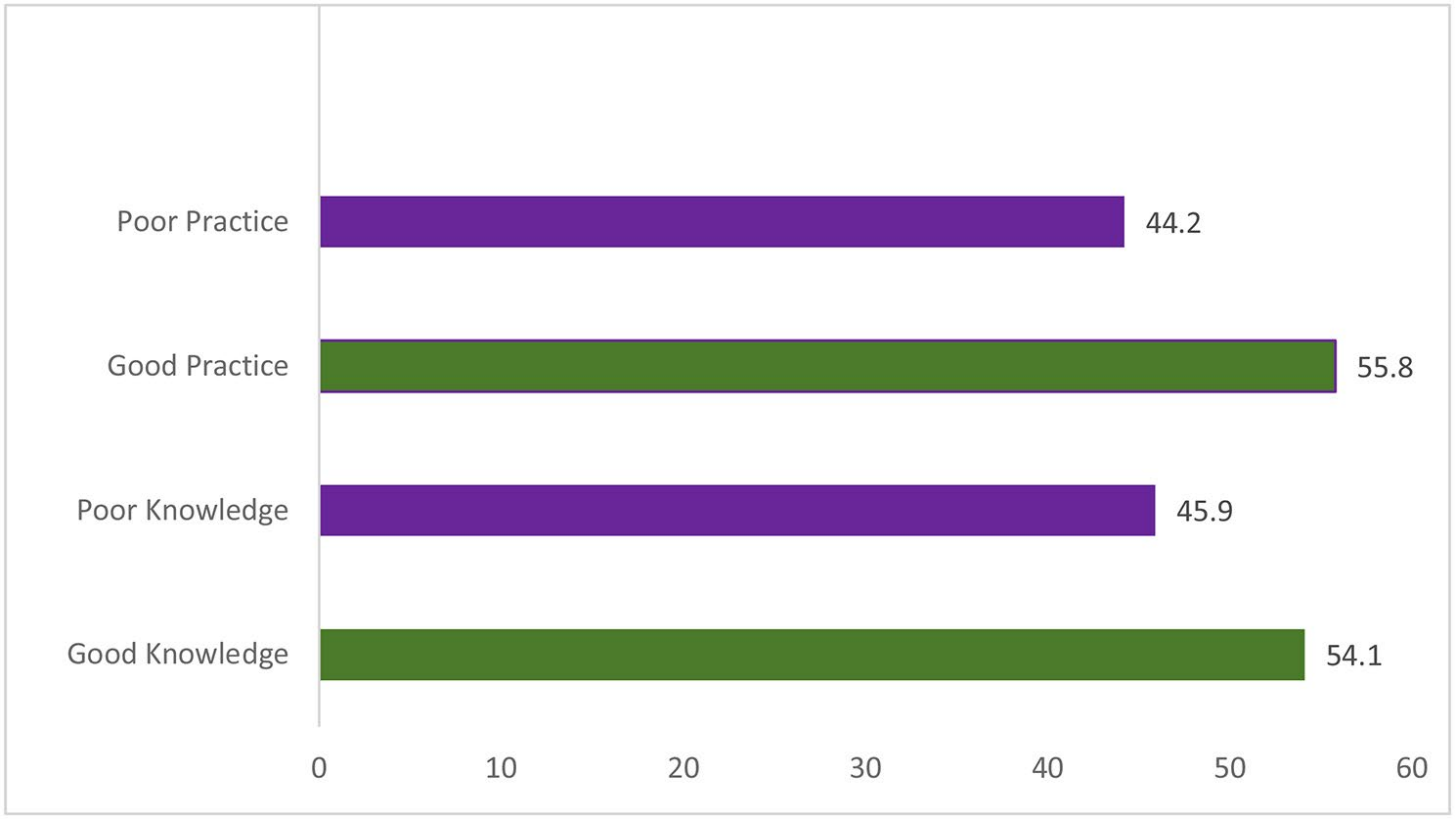
Levels of respondents’ knowledge and practice of oral hygiene

The figure below shows the levels of respondents’ knowledge and practice of oral hygiene. The current study found that a high proportion (*n* = 130, 55.8%; and *n* = 126, 54.1%) of students had good levels of practice and knowledge respectively (Fig. 1).

### Factors associated with the practice of oral hygiene among students

The table below presents the distribution of participants based on their oral hygiene practices and various demographic variables. Pearson’s Chi-square test revealed a significant relationship between the class or education level of students and the practice of oral hygiene (χ2 = 17.36, *p* < 0.001). However, other demographic variables, such as age, sex, ethnicity, religion, occupation of parents/guardian, level of education of parents/guardian, and level of knowledge of oral hygiene did not show any significant association with oral hygiene practice (Table 4).

**Table 4:** Factors associated with the Practice of Oral Hygiene among students

Variable	Practice of Oral Hygiene		Chi-square (χ^2^)	p-value
	Poor Practice 103 (44.2%)	Good Practice 130 (55.8%)		
**Age group (Years)**			1.31	0.520
11–13 years	20 (19.4)	18 (13.9)		
14–16 years	72 (69.9)	97 (74.6)		
17–19 years	11 (10.7)	15 (11.5)		
**Sex**			0.32	0.570
Male	45 (43.7)	52 (40.0)		
Female	58 (56.3)	78 (60.0)		
**Ethnicity**			0.35	0.949
Akan	53 (51.5)	63 (48.5)		
Ga/Adamgbe	17 (16.5)	25 (19.2)		
Ewe	20 (19.4)	26 (20.0)		
Others	13 (12.6)	16 (12.3)		
**Religion**			0.62	0.733
Christianity	94 (91.3)	120 (92.3)		
Islam	7 (6.8)	9 (6.9)		
Traditionalist	2 (1.9)	1 (0.8)		
**Class or education level**			17.36	**< 0.001**
JHS 1	35 (34.0)	28 (21.5)		
JHS 2	39 (37.9)	30 (23.1)		
JHS 3	29 (28.2)	72 (55.4)		
**Occupation of Parents/Guardian**			7.95	0.093
Farmer	12 (11.7)	5 (3.9)		
Trader	55 (53.4)	70 (53.9)		
Government Worker	25 (24.3)	31 (23.9)		
Others	7 (6.8)	19 (14.6)		
Unemployed	4 (3.9)	5 (3.9)		
**Level of education of Parents/Guardian**			1.70	0.637
No Formal Education	13 (12.6)	13 (10.0)		
Primary	14 (13.6)	12 (9.2)		
Secondary	41 (39.8)	56 (43.1)		
Tertiary	35 (34.0)	49 (37.7)		
**Level of Knowledge**			2.28	0.131
Poor Knowledge	53 (51.5)	54 (41.5)		
Good Knowledge	50 (48.5)	76 (58.5)		

## Discussion

Oral hygiene is intricately linked to an individual’s attitude, behaviour, and level of understanding regarding it. Possessing accurate knowledge about oral hygiene plays a crucial role in promoting improved oral care habits [21]. The present study assessed the knowledge and practice of oral hygiene among students as well as the factors associated with their practice. Findings from the study revealed that more than half 126 (54.1%) of the students had a good level of knowledge regarding their oral health. This finding is consistent with studies conducted in Croatia, Saudi Arabia, Zambia and Uganda which found a good level of knowledge among their studied populations [12, 20, 23, 24]. This finding is quite promising as it suggests that a substantial number of students have acquired valuable information about oral hygiene, possibly through educational initiatives, awareness campaigns, or proactive efforts to learn about proper dental care.

Conversely, this study’s finding is inconsistent with a study done in India [22], which reported a lower proportion of knowledge among the respondents. The lower reported knowledge in this study could be attributed to differences in the study population’s demographic characteristics, educational background, access to oral health information, or cultural beliefs and practices regarding oral hygiene. Additionally, contextual factors such as socioeconomic status, healthcare infrastructure, and oral health literacy initiatives within the study setting may also influence the observed differences in knowledge levels.

When asked what the main cause of tooth decay is, 29 (12.5%) of the respondents reported eating sugary foods as the main contributing factor to tooth decay. This suggests that while a minority of participants acknowledged the link between sugary foods and dental decay, there may be varying perceptions or awareness levels regarding this association among the surveyed population. This finding corroborates with previous studies conducted in Saudi Arabia and Nigeria, which also reported similar observations regarding the limited awareness of the link between sugary foods and dental decay [25, 26]. The consistency in findings across these different studies, including the current one, maybe due to shared cultural dietary habits that prioritize sugary foods, as well as inadequate education or public awareness campaigns on the negative effects of excessive sugar consumption.

Unlike the current study, studies done in Pakistan and southern Saudi Arabia revealed a higher proportion of respondents who demonstrated a better understanding of the relationship between tooth decay and sweet food substances [27, 28]. These findings emphasize the importance of tailoring oral health education programs to address specific misconceptions and knowledge gaps about the causes of tooth decay and effective oral hygiene practices. Cultural, regional, and educational factors can significantly influence perceptions about dental health, underscoring the need for context-specific approaches to oral health promotion and disease prevention.

Regarding how frequently to visit a dentist, a significant majority of the respondents 129 (55.4%) reported visiting the dentist at least once every six months is important. This finding aligns with previous studies conducted in Zambia and Zagreb [23, 29] which also observed similar trends among the respondents regarding the frequency of dental visits. The consistent results suggest that regular dental check-ups every six months are crucial in maintaining optimal oral health and preventing dental issues. Regular dental visits allow for early detection of potential problems, timely treatment, and personalized oral health guidance, all of which contribute to better overall dental well-being and a healthier smile [6].

In terms of the practice of oral hygiene, the current study revealed that a significant majority of the respondents, specifically 130 (55.8%) respondents, demonstrated a commendable level of oral hygiene practices. In comparison, this finding aligns with prior studies conducted in Nepal, India and Nigeria [30–33], suggesting a consistent trend of positive oral hygiene behaviours among participants across these diverse regions. The convergence of results emphasizes the universal significance of oral health awareness and education in fostering good oral care habits on a global scale.On the contrary, the current study contradicts the findings from research conducted in Malaysia, Saudi Arabia and southern Ethiopia [21, 28, 34], which reported poor oral hygiene practices among their respondents. This disparity in results underscores the importance of considering regional and cultural factors in understanding oral health behaviours and highlights the need for specific interventions to address oral hygiene challenges in different populations.

The study also revealed that a majority of the respondents, specifically 152 (65.2%) respondents, brushed their teeth two or more times a day, while 77 respondents (33.1%) reported using dental floss regularly. Notably, these findings align with previous studies conducted in South Africa, Kenya and Nigeria [33, 35, 36], which also observed similar findings of frequent toothbrushing and dental floss usage among participants in those regions. The alignment of findings across these studies could be attributed to access to oral hygiene products and peer influence or social networks. This suggests a regional trend in oral hygiene practices, highlighting a shared emphasis on regular toothbrushing and dental floss usage among participants in these regions. In contrast, this study differs from research conducted in Nepal, Ethiopia, and Burkina Faso [30, 37, 38], where a higher proportion of respondents reported brushing their teeth less than twice a day. Unlike the current findings, these studies observed a trend of lower toothbrushing frequency among participants in those regions. One possible explanation is the variations in socioeconomic factors, including access to oral hygiene products and dental care services, which may have influenced respondents’ ability to maintain regular toothbrushing routines. However, this underscores the importance of context-sensitive oral health initiatives to address oral hygiene disparities and promote better dental care practices in diverse communities.

Further, the study made an interesting discovery that a significant majority of respondents 188 (80.7%), reported using fluoridated toothpaste during brushing. This finding aligns with prior research conducted in other settings indicating a consistent trend of utilizing fluoridated toothpaste among participants in different studies [30, 38]. The widespread use of fluoridated toothpaste highlights its acceptance as a valuable oral care product and underscores its role in promoting dental health and preventing tooth decay.

Ultimately, the study explored the factors influencing oral hygiene practices and found a significant association with the class or education level of respondents (χ2 = 17.36, *p* < 0.001). This result indicates that the class or education level of students had a considerable influence on their oral hygiene habits. Similarly, prior studies conducted in Burkina Faso and Nigeria [38–40] also reported a similar observation, highlighting the role of class or education level in shaping oral care behaviours among students. However, our findings diverged from a study conducted in Peru and Ethiopia [34, 41], which reported different results regarding the association between class and oral hygiene practices. The disparity in findings could be attributed to educational systems, and oral health awareness levels among the study populations in regions compared to our research.

## Conclusion

While oral health knowledge among the students was found to be moderate, it is encouraging to note that a majority demonstrated good practice in maintaining their oral hygiene. Regular dental check-ups were recognized as crucial, with a significant number of students visiting dentists at least once every six months. Additionally, we identified a significant association between the class or education level of students and their oral hygiene practices. While other variables did not reach statistical significance, our research serves as a starting point for further investigation and exploration of the various factors that may contribute to oral hygiene practices. Future studies could delve deeper into the influence of socioeconomic status, cultural beliefs, and access to dental care to gain a comprehensive understanding of the complexities surrounding oral health behaviors.

However, the significant relationship between the class or education level of students and good practice of oral hygiene highlights the significance of targeted interventions and educational programs aimed at specific age groups or academic levels. By addressing oral health education in these specific contexts, we may be able to make a more significant impact on improving oral hygiene practices among the population under study. Overall, our study reinforces the significance of oral health education and awareness campaigns, especially in school settings, to improve knowledge, attitudes, and behaviours related to oral hygiene. 

### Implications for policy and practice

The present study highlights the importance of incorporating comprehensive oral health education programs in school curricula. By integrating oral health topics into the educational curriculum, students can gain valuable knowledge about oral hygiene, dental care practices, and the importance of regular dental check-ups. Governments and educational institutions should collaborate to design age-appropriate and culturally relevant oral health education materials and initiatives. Additionally, healthcare policymakers should focus on promoting regular dental check-ups among schoolchildren by advocating for accessible and affordable dental care services. Initiatives like school-based dental clinics or mobile dental units can be introduced to provide preventive and basic dental care services to students. Nonetheless, policymakers should conduct further research to identify specific barriers and challenges faced by different regions in maintaining good oral health. Targeted interventions, tailored to the unique cultural, social, and economic factors of each region, can be developed to address these disparities and improve oral care practices. By incorporating these implications into policy and practice, Ghana can work towards improving the oral health of its younger population, promoting a culture of good oral hygiene habits, and ultimately reducing the burden of oral diseases in the country.

### Limitations and strengths

Despite the valuable insights gained from this study, there are some limitations that should be acknowledged. The sample size of 233 participants may be relatively small to represent the entire population of JSS students in Koforidua. A larger sample size would have provided a more robust and representative view of the students’ oral hygiene practices. The study used a multistage sampling technique, which may introduce sampling bias. For instance, if certain schools or classes were not included in the sample, it might not fully represent the diversity of the population and could influence the results. However, despite these limitations, the study has several notable strengths. The study reported a 100% response rate from the participants included in the sample, indicating a high level of cooperation and engagement from the students. This strengthens the reliability of the data collected. The questionnaire used in the study was pre-validated and adopted from previous studies, ensuring the reliability and validity of the data collected. This enhances the comparability of the findings with other similar studies. Additionally, the study concludes with implications for policy and practice, providing valuable recommendations for promoting better oral hygiene habits among JSS students. These practical implications can inform the development of targeted oral health education programs.

## Data Availability

All data generated or analyzed during this study are included in this published article.

## Abbreviations

JHS: Junior High School
PI: Principal Investigator
WHO: World Health Organization
